# Evaluation of foreign national cases applied to Tokat Gaziosmanpasa University Hospital Forensic Medicine Department, 2014–2022

**DOI:** 10.1007/s00414-024-03246-8

**Published:** 2024-05-22

**Authors:** Selçuk Çetin, Mete Gedikbaş, Şule Sinem Gedikbaş

**Affiliations:** 1 Department of Forensic Medicine, Faculty of Medicine, Tokat Gaziosmanpasa University, Tokat, Turkiye Turkey; 2 Department of Orthopedics and Traumatology, Turhal State Hospital, Tokat, Turkiye Turkey

**Keywords:** Foreigners, Forensic medical report, Forensic medicine, Refugee

## Abstract

**Introduction/aim:**

Turkey has experienced a heavy migration burden in recent years due to its location and benevolent policies. This study aimed to retrospectively examine and discuss the reports prepared for foreign nationals who requested assistance at the Forensic Medicine Department of Tokat Gaziosmanpaşa University Hospital in Tokat, which is located in the Middle Black Sea Region of Turkey, between 2014 and 2022.

**Materials and methods:**

This study retrospectively evaluated reports prepared between 2014 and 2022 in the outpatient clinics of Forensic Medicine at Tokat Gaziosmanpaşa University Hospital.

**Results:**

Based on the files reviewed, 219 cases were included in the study, of which 70.8% (*n* = 155) were male and 29.2% (*n* = 64) were female. Among the 75 cases referred by judicial authorities, 34.6% (*n* = 26) involved assault, 28% (*n* = 21) involved determination of the ability to understand the legal significance and consequences of the act, 16% (*n* = 12) involved traffic accidents, 8% (*n* = 6) involved poisoning, 9.3% (*n* = 7) involved abuse, and 4% (*n* = 3) involved age determinations.

**Discussion:**

Problems with immigrants, which have always been a reality due to Turkey’s location on migration routes, have increased significantly in recent years. For this reason, we believe that studies with multicenter and larger series should be conducted to determine the current situation that foreigners create for themselves and Turkey to facilitate necessary arrangements, determine proposed solutions, increase the quality of services offered, and develop plans for the future.

**Supplementary Information:**

The online version contains supplementary material available at 10.1007/s00414-024-03246-8.

## Introduction

The concept of a foreigner was defined at the Geneva meeting of the Institute of International Law in 1892 as “a person residing in the territory of a State who does not have the right to claim the nationality of that State” [1]. Depending on the classification of the various categories under the term “alien,” stateless persons, persons with multiple nationalities, refugees, and asylum seekers may also be considered aliens [2]. Thousands of people leave their homelands to achieve a better quality of life. In fact, the concept of migration, defined as the pursuit of people’s needs, is the displacement of people to settle in the future [3]. According to Article 3 of the Turkish Citizenship Law (TCL) 2009, no. 5901 and the Law on Foreigners and International Protection (FIPL) 2013, no. 6458, a foreigner is defined as a person who has no civic ties with the state of the Republic of Turkey. Therefore, anyone who does not have Turkish citizenship is a foreigner in Turkey [1].

Although migration is a common reality in geographies that support transition, sudden migration causes major change [4]. According to the United Nations High Commissioner for Refugees (UNHCR), Turkey became the world’s largest host country for refugees in March 2015 [5]. Since 2011, migration has become a major issue in Turkey, and public service ethics have become an area of concern. In addition to the safety of those who have come to Turkey through immigration, the safety of those living in Turkey has also become an important concern.

Considering that Turkey has one of the highest numbers of foreign nationals in the world and that the number of people immigrating there increases every year, it is necessary to reveal the problems experienced by foreign nationals residing in Turkey, such as healthcare, education, working life, and accommodations. It is crucial not only to solve these problems but also to evaluate them in terms of incidents that are reflected in judicial records, such as crimes, and whether immigrants are victims or perpetrators of traffic accidents or work accidents.

This study aimed to retrospectively examine and discuss the reports prepared for foreign nationals who requested assistance at the Forensic Medicine Department of Tokat Gaziosmanpaşa University Hospital in Tokat, which is located in the Middle Black Sea Region of Turkey, between 2014 and 2022.

## Materials and methods

Ethics committee approval (83116987-775) was obtained from our institution before beginning the study. We retrospectively analyzed the records of foreign nationals who reported to forensic medicine department of Tokat Gaziosmanpasa University hospital between 2014 and 2022. For those who did not speak Turkish, the report was kept with translators appointed by the court.

The hospital records included age, sex, the subject of the report, the event that caused the injury, a description of the injury, which body part was injured in cases reported after traffic accidents, and crimes committed by delinquent children, including whether they committed the crime alone or in groups and the legal status of the crime.

We also evaluated the parameters regarding their ability to recognize the meaning and consequences of their crime to manage their behavior, the crime scene, the victim–offender relationship, and the type of abuse in cases of sexual abuse.

### Statistical analysis

The data were analyzed using descriptive statistics. Qualitative variables were expressed as number (n) and percentage (%), whereas quantitative variables were presented as number, minimum, maximum, mean, and standard deviation (mean ± Ss). We used IBM Statistical Package for the Social Sciences 29 to analyze the data.

## Results

This study included 219 cases of foreign nationals who presented to our clinic between 2014 and 2022, of which 70.8% (*n* = 155) were male and 29.2% (*n* = 64) were female. The mean age was 24 (4–62 years), with 37.6% (*n* = 82) children and 62.4% (*n* = 136) adults. The demographic data of the cases are detailed in Table 1.

**Table 1:** Demographic characteristics of the foreign nationals who presented to our outpatient clinic

	219 cases
Age (year)	24.42 ± 13.79 (4–62)
Age group	
Child	82
Adult	136
Sext (Female/Male)	64 / 155
Working status (Regular/Irregular)	21 / 198
Post-migration time	
<5 years	157
>5 years	62
Turkish citizenship (yes/no)	2 / 217

Of the 75 cases referred by judicial authorities, 34.6% (*n* = 26) were for assault, 28% (*n* = 21) were determinations of the ability to understand the legal significance and consequences of the act, 16% (*n* = 12) were traffic accidents, 8% (*n* = 6) were poisonings, 9.3% (*n* = 7) were abuse, and 4% (*n* = 3) were age determinations. Additionally, 144 cases presented to our outpatient clinic for physical disability determination after a traffic accident. Details the reasons for admission and the injury ranges of the cases showed in Table 2.

**Table 2:** Reasons for admission and places of injury of the foreign nationals who presented to our outpatient clinic

	219 cases
**Admission type**	
Traffic accident or assault	182
Poisoning	6
Abusement	7
Ascertain the ability to recognise the legal significance and consequences of action	21
Age determination	3
Site of injury	
Head trauma	51
Extremity injury	93
Thorax injury	9
Abdominal injury	11
Other	18

There were 26 cases investigated for assault, of which 4 were female and 22 were male, with an average age of 28.5 years (10–55 years). Six of our cases were illiterate, nine had a primary school degree, six had a secondary school degree, and one had a university degree. The educational status of the remaining four people was unknown. Only four of the cases examined for assault were engaged in regular work.

In the analysis of the 21 cases examined in our outpatient clinic, it was found that 66.7% (*n* = 14) were reported for fighting and 23.8% (*n* = 5) were reported for theft. It was found that 61.9% (*n* = 13) of these cases were involved in the crime alone, and 31.8% (*n* = 8) were involved as part of a group. As a result of the investigation and evaluation, 85% (*n* = 17) of these children reported that their ability to recognize and manage the legal significance and consequences of their offenses was improved (Table 3).

**Table 3:** Data on delinquent foreign youth and the type of crime they are alleged to have committed, the manner in which they were involved in the crime, and their ability to recognise the legal significance and consequences of the crime

	219 cases
Type of crime	
Fight	14
Theft	5
Other	2
Manner of committing a crime.	
Individual	13
Group	8
Ability to recognise the legal significance and consequences of action	
Improved	17
Null	4

Cases admitted to our clinic due to sexual abuse accounted for 9.3% (*n* = 7) of all cases. It was noted that the time of admission for 3 of the cases was 3–10 days after the event, while the time of admission for an additional 3 was after 10 days. In four of these cases, the perpetrator was known to the victim, while in two cases, the victim stated that they had been abused by a person unknown to them.

## Discussion

The socioeconomic, cultural, and political structure of the country to which they have immigrated and the problems that immigrants have faced or are facing in the country to which they have immigrated contribute to increased litigation [6]. The view that the crime rate among foreigners is high is widespread. Research on this topic indicates that a large portion of the public believes that immigrants commit more crimes than natives [7]. One of the main causes of increases in violent crime following domestic wars is the development of a culture or approach that legitimizes violence [8]. There are also many behavioral problems related to trauma, especially in people who find themselves in a violent and warlike environment and then leave it. It has been observed that this leads to the development of negative behaviors, such as anger and aggression, poor academic performance, and substance abuse [9]. It is inevitable that negative events will occur as a result of these behaviors.

In Gaziantep, where more than 50,000 Syrians live, it was found that the number of crimes involving Syrians in 2015 was 1,926, of which 248 were negligent injuries, 238 were intentional injuries, 95 were thefts, 14 were robberies, 59 were threats, and 26 were insults. In Kahramanmaraş, where about 85,000 Syrians live, 985 crimes were registered by Syrians, of which 241 were negligent assaults, 183 were intentional assaults, and 59 were thefts. In Kilis, where 16,734 Syrians live, 3,400 were involved in public order, and the crime rate originating from Syrians reached 30% in the city [10]. According to data from the Migration Management Directorate, there are 3,854 immigrants living in the province of Tokat, of which 1,179 are Syrians. Based on these data, it was found that the crime rate is in line with previous studies in Turkey.

A 2012 study of foreigners living in the Netherlands and Belgium by Keygnaert et al. reported that about 47% of people in those countries had been exposed to physical violence during their time in those countries, although most had sustained non-life-threatening injuries [11]. In another study conducted in Italy on 503 people, it was found that about half had been exposed to violence by relatives or foreigners [12]. In a study conducted by Atalay et al. in Turkey on asylum seekers who made emergency applications in Hatay province, people under 18 years of age were examined, with 54.7% of the cases due to gunshot wounds [13]. In accordance with the literature, the vast majority of the 219 cases examined in our study involved various injuries.

Clinical forensic medicine was first applied in the United Kingdom in 1951 and has gained prominence and widespread use worldwide over the past 20 years [14]. ​Chaudhary et al. reported in their study that the results of examinations conducted by a forensic medicine specialist were more detailed, understandable, and results-oriented [15]. The evaluation of individuals involved in traffic accidents has become a daily routine in many forensic medical institutions. Along with the increase in disability reports sent out by the courts, individual requests from individuals to determine their own degree of physical disability are also increasing daily [16, 17]. In our study, the majority of the cases consisted of individuals who were injured after a traffic accident and were used individually to determine the degree of physical disability. We believe that in Turkey, where the number of vehicles and traffic accidents is increasing, necessary legal and regulatory measures should be taken, training programs should be organized to raise awareness of each individual, and necessary control mechanisms should be actively implemented.

Reports prepared by Çukurova University Faculty of Medicine, Department of Forensic Medicine to determine physical disability found lower extremity injuries in 2,160 (56.3%) cases, upper extremity injuries in 1,010 (26.3%) cases, head injuries in 690 (17.9%) cases, 172 (4.5%) cases, thoracic injuries in 122 (3.1%) cases, abdominal injuries in 122 (3.1%) cases, and vertebral injuries in 464 (12%) cases [18]. In our study, most cases were admitted because of injuries to the extremities.

Without a job that provides a regular income, there is a risk that daily life cannot be sustained. For this reason, crime rates may be higher to meet the basic needs of people with low income levels. In studies conducted in 28 European countries and in countries with high migrant populations, such as Malaysia, Mexico, and Pakistan, unemployment was found to be positively correlated with crime [19–23]. Studies have also shown that the crime rate is higher among people under 24 years of age [24, 25]. Additionally, a negative correlation has been found between education levels and crime rates [22, 26]. Similarly, we found that the educational level of our cases examined for assault was low and that most of them had not held regular employment during the first three decades of their lives.

It has been observed that people who have experienced war later develop tension, frightfulness, restlessness, anger outbursts, and aggressive behavior. In addition, a child who has been exposed to a conflict situation may be angry with the warring parties as well as with their parents and relatives because they cannot adequately protect themselves from what is happening or provide adequate options. The anger in this situation may be directed toward different parties and people [27]. Because conflict is common among foreigner children before or during flight, these children may lose their moral perspective and believe that looting and stealing are not the same thing or that killing may be justified for political reasons [28]. Foreigner children in particular are exposed to both environmental and domestic violence, so they see violence as a natural means of self-defense and self-expression [29]. It is said that asylum seekers suffer more from mental health problems for reasons such as leaving their homes, leaving their loved ones behind, the language, culture, and attitudes of the new country, the loss of family members, and interruptions in their education. Studies of Syrian refugees also support this information, showing that foreigners feel lonely, unhappy, unappreciated, excluded, and unsafe [30]. In studying the characteristics of children involved in delinquency, we found that most of them were aware that their actions constituted a criminal offense. We think that this situation is due to the environment and psychological burnout. To prevent these issues among foreigner children, rehabilitation programs should be implemented to help them adapt to a new social environment. In addition, forced migrations must be controlled and recorded, all humanitarian support must be provided throughout the adaptation processes, and training and adaptation programs must be systematically created. These programs must be auditable and monitored by international organizations.

Adapting to an environment dominated by a new culture, language, and values takes time and presents many difficulties [30]. In addition to crimes such as theft and fights, violent crimes such as sexual abuse are inevitable. A study of persons under the age of 18 who were subjected to sexual abuse in Muğla found that 2.9% of the victims were foreign nationals, while this rate reached 9.8% in a larger series that included all age groups [31, 32]. Araujo et al.’s systematic review of the prevalence of sexual violence among foreigners found that the vast majority of cases of sexual violence were perpetrated by close partners (55%), acquaintances, and relatives [33]. In our study, the rate of cases sent to our clinic for sexual abuse was 9.3% of all cases. Of those cases, five knew the abuser, including one who was a family member, while two claimed to have been abused by a person unknown to them.

The limitations of this study include the limited number of cases compared to other study groups conducted in our country and abroad and the retrospective design. The fact that the study was based on data obtained in a single center using standardized examination forms was the strength of this study. Using the form we used in the study in other centers and applying the case history will support our study.

## Conclusion

The number of foreign nationals in Turkey, including immigrants, foreigners, and asylum seekers, continues to increase, despite the country being above capacity. We are of the opinion that multicenter and comprehensive studies with a greater number of cases regarding foreign nationals, which has been the reality of our country for an extended period, can provide more accurate data on foreign nationals and thus offer optimal solution proposals. It is known that as well as the problems caused by uncontrolled or intense migrations on the settled society, asylum seekers also experience many problems and difficulties in the adaptation process to the cultures, legal systems, social behavioral habits and many other settled situations of the places they migrate to. For this reason, forced migrations must be controlled and recorded, all humanitarian support must be provided throughout the adaptation processes, and training and adaptation programs must be systematically created. These programs must be auditable and monitored by international organizations. Approaches aimed at stopping migration in only one region by international institutions and organizations do not prevent this problem from affecting wider geographies. Situations that are normal in the country where foreigners come from may not only be crimes in the Turkish penal code, but also create legal and sociocultural problems. Training processes regarding these should be carried out in a controlled manner. Failure to follow the legal process may make people more likely to commit crimes in the future.

### Electronic supplementary material

Below is the link to the electronic supplementary material.

Supplementary Material 1

Supplementary Material 2
