# Supplementary material for: Evaluation of foreign national cases applied to Tokat Gaziosmanpasa University Hospital Forensic Medicine Department, 2014–2022
Source: Int J Legal Med. 2024 May 22;138(5):2015–20. doi: 10.1007/s00414-024-03246-8 (PMC11306730; doi:10.1007/s00414-024-03246-8)
Supplement: Supplementary file 2 — Supplementary Material 2 [file 414_2024_3246_MOESM2_ESM.pdf]

T.C.
TOKAT GAZİOSMANPAŞA ÜNİVERSİTESİ
TIP FAKÜLTESİ DEKANLIĞI
Klinik Araştırmalar Etik Kurulu

Sayı	:	83116987 - 775
Konu	:	Etik Kurul Kararı
Toplantı Tarihi	:	24.11.2022
Toplantı No	:	2022/20
Proje No	:	22-KAEK-264

08.12.2022

Sayın, Dr. Öğretim Üyesi Selçuk ÇETİN

Etik Kurulumuzun 24.11.2022 tarihli toplantısında görüşülen **22-KAEK-264** kayıt numaralı **"Tokat Gaziosmanpaşa Üniversitesi Hastanesi Adli Tıp Polikliniği'ne 2014-2022 Yılları Arasında Başvuran Yabancı Uyruklu Hastaların Değerlendirilmesi"** başlıklı çalışmanız gerekçe, amaç, yaklaşım ve yöntemleri dikkate alınarak incelenmiş ve uygun bulunmuş olup, çalışmanın başvuru dosyasında belirtilen merkezde gerçekleştirilmesinde etik ve bilimsel sakınca bulunmadığına karar verilmiştir.

İlaç ve Biyolojik Ürünlerin Klinik Araştırmaları Hakkında Yönetmeliğin 14-4. maddesi ve yönergemizin 18-3. maddesine göre çalışmanız tamamlandıktan sonra sonuç raporunun tarafımıza en geç 90 gün içerisinde bildirilmesi gerekmektedir.

Bilgilerinizi rica ederim.

Doç.Dr. Muzaffer KATAR
Başkan

Scanned with ACE Scanner
