# Supplementary material for: Evaluation of foreign national cases applied to Tokat Gaziosmanpasa University Hospital Forensic Medicine Department, 2014–2022
Source: Int J Legal Med. 2024 May 22;138(5):2015–20. doi: 10.1007/s00414-024-03246-8 (PMC11306730; doi:10.1007/s00414-024-03246-8)
Supplement: Supplementary file 1 — Supplementary Material 1 [file 414_2024_3246_MOESM1_ESM.pdf]

Selçuk Çetin
Assistant Professor
Tokat Gaziosmanpaşa University, Medical School,
Department of Forensic Medicine, Tokat, Turkey.

07.11.2023

Dear Editor,

We wish to submit an original research article entitled ‘’Evaluation of foreign national cases applied to Tokat Gaziosmanpasa University Hospital Forensic Medicine Department, 2014–2022” for consideration by International Journal of Legal Medicine.

We confirm that this study is original and has not been published elsewhere, nor is it currently under consideration for publication elsewhere.

Migrations, primarily caused by wars and other compulsory reasons, have become one of the most important humanitarian problems in our country and neighboring geography. With these migrations, it becomes inevitable to experience important medicolegal problems regarding both the local people and the refugees. For this reason, we wanted to discuss the data regarding our foreign national cases who applied to our Department, from a medicolegal perspective, in the light of the literature.

We believe that this article is suitable for publication by the International Journal of Legal Medicine because we believe that the findings will contribute to drawing attention to this issue.

We have no conflicts of interest to disclose.

Please address all correspondence concerning this manuscript to me at dr.scetin84@gmail.com

Thank you for your consideration of this manuscript.

Sincerely,

Selçuk Çetin
